# COVID-19 vaccine hesitancy and influencing factors among Chinese hospital staff: a cross-sectional study

**DOI:** 10.1038/s41598-024-55001-z

**Published:** 2024-02-22

**Authors:** Shangyao Li, Jinjuan Hao, Yu Su, Haoran Zhan, Nuo Zhou, Yitong Qiu, Yitong Lu, Ke Sun, Yu Tian

**Affiliations:** 1 School of Public Health, Capital Medical University, 10 You’anmen Outer West 1st Street, Beijing, 100069 China; 2 Hospital Administration Office, Beijing Hospital, National Center of Gerontology, Institute of Geriatrics Medicine, Chinese Academy of Medical Sciences, 1 Dahua Road, Dongdan, Beijing, 100730 China

**Keywords:** COVID-19 vaccines, Vaccine hesitancy, Vaccination willingness, Influencing factors, Disease prevention, Public health

## Abstract

We aimed to investigate the willingness of hospital staff to receive the COVID-19 vaccine and explore the associated factors and reasons of vaccine hesitancy among Chinese hospital staff, which were not yet known. A cross-sectional questionnaire survey was conducted online on the vaccine hesitancy of staff in a grade A tertiary general hospital in Beijing from February 22 to 23, 2023. Univariate and multivariate logistic regression were used to assess associations between potential influencing factors and vaccine hesitancy. A total of 3269 valid respondents were included, and the rate of COVID-19 vaccine hesitancy was 32.67%. Multivariate logistic regression showed that women [1.50 (1.22–1.83)], having high-school education level [1.69 (1.04–2.76)], college degree [2.24 (1.35–3.72)] or graduate degree [2.31 (1.33–4.03)], and having underlying disease [1.41 (1.12–1.77)] were associated with a higher rate of COVID-19 vaccine hesitancy. The main reasons for vaccine hesitancy included doubts for the safety and effectiveness of COVID-19 vaccine and worries in adverse reactions. Hospital staff's willingness to vaccinate COVID-19 vaccine is generally high in the study. Hospitals should spread the knowledge of COVID-19 vaccine through multiple channels to improve the cognition of hospital staff and encourage vaccination based on associated factors.

## Introduction

Novel coronavirus disease (COVID-19) is a global pandemic with the widest impact on human beings in the past centuries, and the life security and health of human beings all over the world are greatly threatened. As of January 25, 2023, the number of positive nucleic acid tests and the positivity rate of the COVID-19 reporting population in each province of China showed a trend of increasing and then decreasing, with the number of positives reaching a peak on December 22, 2022 (6.94 million), after the nationwide cessation of nucleic acid screening for the whole population on December 8, 2022^1^.

Vaccine, as one of the most important public health measures, plays a vital role in dealing with the epidemic of infectious diseases. Vaccination is an important factor in controlling infection rate, severe case rate and mortality rate^2^. The Chinese Government has been always working on ensuring all people eligible for vaccination have access to it, and has actively introduced incentive policies for vaccination, such as free vaccinations for all people, incentive benefit, and first serve for priority groups including those engaged in handling imported cold-chain products and people working in port inspection and quarantine, aviation, public transport, fresh market, medical treatment, and disease control.

However, with the promotion of COVID-19 vaccination, some people are prone to vaccine hesitancy due to kinds of reasons in the world, leading to a decline in vaccine coverage and timeliness^3–7^. Vaccine hesitancy is defined as “delay in acceptance or refusal of safe vaccines despite availability of vaccination services” by the Strategic Advisory Group on Experts (SAGE) Working Group on Vaccine Hesitancy^8^. The World Health Organization named vaccine hesitancy as one of the top ten threats to global health in 2019 and emphasized that the reasons why people chose not to vaccinate were complex^9^. Vaccine hesitancy varies with time, place, type of vaccine and other factors, which are influenced by individual complacency, vaccination convenience and confidence in the vaccine and so on^8^. With volatility of the peak infection period in many places in China, future vaccination has become a big health concern.

Many factors affect decision-making in COVID-19 vaccination, usually involving a trade-off between risks and benefits of vaccination^10^. For medical workers, their high education level and rich clinical practice make them more willing to vaccinate against COVID-19. Moreover, the nature of medical workers’ work determines their high risk of COVID-19 infection^11^. Therefore, medical staff become a group having the priority of vaccination and protection in COVID-19, as well as a main driving force for the public to vaccinate against COVID-19, for example, medical staff's low willingness to recommend or refuse to vaccinate against COVID-19 will significantly affect patients' trust in vaccines, leading to public hesitancy and crisis of confidence^12^. Some studies have investigated reasons for medical staff's hesitancy in the COVID-19 vaccine^13–15^, but there are few studies on Chinese medical workers. In our study, we investigated the vaccine hesitancy of staff in a hospital in Beijing in COVID-19 vaccination and explored influencing factors of vaccine hesitancy, aiming to provide an evidence support for further improving vaccine acceptance and vaccination rate of hospital staff.

## Methods

### Study participants

From February 22 to February 23, 2023, all the hospital staff, including medical staff, researcher, students, etc. of a grade A tertiary general hospital in Beijing which focuses on geriatric medicine and excels in respiratory and critical care medicine were selected to participate an online questionnaire survey, as a part of routine individual monitoring and reporting of infection and vaccination from each department. Based on the reliability (Cronbach’s α = 0.864) and validity (KMO = 0.797) of the questionnaire and a 40% prevalence of COVID-19 vaccine hesitancy in the pilot survey, we estimated the minimum sample size required for the formal survey to be 1282 participants, with 3% margin of error and 20% missing data.

### Data collection

According to people’s knowledge and willingness to vaccinate against COVID-19 and the reference of relevant literature related to vaccine hesitancy^16–18^, an online questionnaire was designed and formed, of which all questions were obligatorily answered by participants in their cellphones with full anonymity and were allowed to be submitted once. The questionnaire comprised four parts: demographics, health status before infection (underlying disease was defined as chronic illnesses, including hypertension, respiratory disease, kidney disease, diabetes, and cancer), COVID-19 vaccination and vaccine hesitancy. Vaccine hesitancy in our study used the definition by the Strategic Advisory Group on Experts (SAGE) Working Group on Vaccine Hesitancy^8^. As a part of the questionnaire, a scale consisting of multi-dimensional questions related to individual attitude and perception was developed, aiming to measure reasons of vaccine hesitancy among hospital staff. Considering the homogeneity and equality of vaccination policy in China, items involving race, religion, and income were excluded. According to Likert's five-level scoring, the attitude and perception to the items of “reason of vaccine hesitancy” was divided into five grades: very disagreeable, disagreeable, unclear, agreeable, and very agreeable, quantified as 1–5 points^19^. After the online questionnaire survey, the quality control was conducted, and questionnaires were excluded as invalid ones if the following situations happened: (1) There were logical errors in answering the questionnaire; (2) Participants were not vaccinated with COVID-19 vaccine due to contraindications of COVID-19 vaccination.

### Statistical analysis

Demographics and status of vaccine hesitancy were described by frequencies and proportions. The Chi-square test and Fisher’s exact test were used for comparison of categorical variables. Univariate logistic regression was used to preliminarily analyze potential influencing factors associated with vaccine hesitancy. To further adjust for potential confounders of vaccine hesitancy, a multivariate logistic regression analysis was performed, using vaccine hesitancy (binary with Y/N) as the dependent variable, and using gender, age, education degree, occupation, professional title, health status before infection, and vaccination booster doses as the independent variables. Moreover, 196 participants, half of whom were doctors and the other half nurses, were matched using propensity score matching (PSM) of 1:1 k-nearest neighbor matching. None of the variables utilized in the final analysis contained missing data. A two-sided *P*-value < 0.05 was considered statistically significant. All analyses were performed using SPSS 26.0 and R 4.2.3. 

### Ethics approval and consent to participate

The study was approved by the Ethical Review Committee of Beijing Hospital (2023BJYYEC-044-01) and all research was performed in accordance with the relevant guidelines and regulations. All the respondents were informed of the purpose of the study and volunteered to participate, and informed consents were virtually acquired before participating in the survey.

## Results

### Characteristics and vaccine hesitancy of participants

In the online survey, a total of 3679 participants received the survey questionnaire and 3442 of them fully responded, giving a full response rate of 93.56%. The remaining participants either chose to skip or closed the questionnaire before submission. Among 3442 respondents, 3269 questionnaires (94.97%) were valid and included after quality control, of which there were 2358 women (72.13%). The characteristics and health status of respondents were shown in Table 1.

**Table 1:** Demographic characteristics and vaccine hesitancy in COVID-19 of hospital staff.

Characteristics	Total	Vaccine acceptance	Vaccine hesitancy	Rate of hesitancy	*χ*^2^	*P*
	N (%)	n (%)	n (%)	%		
Gender					39.570	< 0.001
Men	911 (27.87)	689 (31.30)	222 (20.79)	24.4		
Women	2358 (72.13)	1512 (68.70)	846 (79.21)	35.9		
Age					30.305	< 0.001
< 30 years	900 (27.53)	587 (26.67)	313 (29.31)	34.8		
30–44 years	1274 (38.97)	808 (36.71)	466 (43.63)	36.6		
45–59 years	1009 (30.87)	742 (33.71)	267 (25.00)	26.5		
≥ 60 years	86 (2.63)	64 (2.91)	22 (2.06)	25.6		
Education degree					66.588	< 0.001
Below high school	297 (9.09)	254 (11.54)	43 (4.03)	14.5		
High school	195 (5.97)	151 (6.86)	44 (4.12)	22.6		
College	1825 (55.82)	1157 (52.57)	668 (62.54)	36.6		
Graduate	952 (29.12)	639 (29.03)	313 (29.31)	32.9		
Occupation					73.90	< 0.001
Doctor	576 (17.62)	400 (18.17)	176 (16.48)	30.6		
Medical technician	333 (10.19)	212 (9.63)	121 (11.33)	36.3		
Nurse	1068 (32.67)	645 (29.31)	423 (39.60)	39.6		
Researcher	91 (2.78)	64 (2.91)	27 (2.53)	29.7		
Administrator	202 (6.18)	139 (6.32)	63 (5.90)	31.2		
Logistician	208 (6.36)	146 (6.63)	62 (5.81)	29.8		
Outsourcing worker	441 (13.49)	363 (16.49)	78 (7.30)	17.7		
Medical student	216 (6.61)	147 (6.68)	69 (6.46)	31.9		
Academic student	134 (4.10)	85 (3.86)	49 (4.59)	36.6		
Professional title					36.103	< 0.001
None	1015 (31.05)	730 (33.16)	285 (26.68)	28.1		
Junior	756 (23.12)	473 (21.49)	283 (26.50)	37.4		
Intermediate	1035 (31.66)	657 (29.85)	378 (35.39)	36.5		
Sub-senior	266 (8.14)	187 (8.50)	79 (7.40)	29.7		
Senior	197 (6.03)	154 (7.00)	43 (4.03)	21.8		
Health status before infection					7.108	< 0.01
Without underlying disease	2818 (86.20)	1922 (87.32)	896 (83.90)	31.8		
With underlying disease	451 (13.80)	279 (12.68)	172 (16.10)	38.1		
Vaccination booster doses					227.933	< 0.001
None	91 (2.78)	37 (1.68)	54 (5.06)	59.3		
1 dose	46 (1.41)	18 (0.82)	28 (2.62)	60.9		
2 doses	470 (14.38)	197 (8.95)	273 (25.56)	58.1		
3 doses	2196 (67.18)	1595 (72.47)	601 (56.28)	27.4		
≥ 4 doses	466 (14.25)	354 (16.08)	112 (10.48)	24.0		

### Influencing factors of vaccine hesitancy

1068 (32.67%) of the 3269 hospital staffs in this survey have reported vaccine hesitancy. The results of the Chi-square test and Fisher’s exact test showed that there was significant difference in vaccine hesitancy between gender, age groups, educational degree, occupations, professional titles, having underlying diseases or not, and booster vaccination doses (all *P* values < 0.01) (Table 1).

Univariate logistic regression showed that women staff (OR: 1.74, 95% CI: 1.46–2.07), educational level of high school [1.72 (1.08–2.74)], having college degree [3.41 (2.44–4.78)], graduate degree [2.89 (2.04–4.11)], nurse [1.49 (1.20–1.85)], junior and intermediate professional titles [1.53 (1.25–1.87) and 1.47 (1.22–1.78), respectively] and hospital staff with underlying diseases [1.32 (1.08–1.63)] were associated with higher COVID-19 vaccine hesitancy (all *P* values < 0.001). In contrast, the middle-aged (45–59 years old) [0.68 (0.56–0.82)], outsourcing worker [0.49 (0.36–0.66)] and hospital staff vaccinated with 3 or 4 booster doses [0.26 (0.17–0.40); 0.22 (0.14–0.35)] were associated with lower vaccine hesitancy (Table 2).

**Table 2:** Univariate and multivariate logistic regression of COVID-19 vaccine hesitancy among hospital staff.

Characteristics	Univariate analysis		Multivariate analysis	
	OR (95% CI)	*P*	OR (95% CI)	*P*
Gender				
Men	Ref		Ref	
Women	1.74 (1.46–2.07)	< 0.001	1.50 (1.22–1.83)	< 0.001
Age				
< 30 years	Ref		Ref	
30–44 years	1.08 (0.91–1.29)	0.389	1.10 (0.85–1.43)	0.450
45–59 years	0.68 (0.56–0.82)	< 0.001	0.99 (0.72–1.36)	0.949
≥ 60 years	0.65 (0.39–1.07)	0.087	1.32 (0.72–2.42)	0.373
Education degree				
Below high school	Ref		Ref	
High school	1.72 (1.08–2.74)	0.022	1.69 (1.04–2.76)	0.034
College	3.41 (2.44–4.78)	< 0.001	2.24 (1.35–3.72)	0.002
Graduate	2.89 (2.04–4.11)	< 0.001	2.31 (1.33–4.03)	0.003
Occupation				
Doctor	Ref		Ref	
Medical technician	1.30 (0.98–1.73)	0.074	1.26 (0.90–1.77)	0.180
Nurse	1.49 (1.20–1.85)	< 0.001	1.31 (0.95–1.80)	0.095
Researcher	0.96 (0.59–1.56)	0.865	0.81 (0.49–1.36)	0.427
Administrator	1.03 (0.73–1.46)	0.867	0.94 (0.64–1.38)	0.741
Logistician	0.97 (0.68–1.36)	0.841	1.02 (0.67–1.54)	0.945
Outsourcing worker	0.49 (0.36–0.66)	< 0.001	0.83 (0.49–1.41)	0.491
Medical student	1.07 (0.76–1.49)	0.706	0.92 (0.60–1.40)	0.680
Academic student	1.31 (0.88–1.94)	0.179	1.07 (0.66–1.73)	0.780
Professional title				
None	Ref		Ref	
Junior	1.53 (1.25–1.87)	< 0.001	0.95 (0.72–1.26)	0.727
Intermediate	1.47 (1.22–1.78)	< 0.001	0.95 (0.70–1.30)	0.752
Sub-senior	1.08 (0.80–1.46)	0.602	0.85 (0.56–1.28)	0.423
Senior	0.72 (0.50–1.03)	0.072	0.63 (0.38–1.03)	0.064
Health status before infection				
Without underlying disease	Ref		Ref	
With underlying disease	1.32 (1.08–1.63)	0.008	1.41 (1.12–1.77)	0.004
Vaccination booster doses				
None	Ref		Ref	
1 dose	1.07 (0.52–2.20)	0.863	1.24 (0.59–2.60)	0.568
2 doses	0.95 (0.60–1.50)	0.824	1.03 (0.64–1.63)	0.916
3 doses	0.26 (0.17–0.40)	< 0.001	0.30 (0.20–0.47)	< 0.001
≥ 4 doses	0.22 (0.14–0.35)	< 0.001	0.22 (0.14–0.36)	< 0.001

Multivariate logistic regression further showed that women staff [1.50 (1.22–1.83)], having higher degree of education [High school: 1.69 (1.04–2.76), college: 2.24 (1.35–3.72), graduate: 2.31 (1.33–4.03), all *P* values < 0.05, trend *P* value = 0.014] and having underlying diseases [1.41 (1.12–1.77)] was associated with a higher vaccine hesitancy. However, having received 3 or ≥ 4 booster doses [0.30 (0.20–0.47) and 0.22 (0.14–0.36), respectively] were associated with a lower vaccine hesitancy (Table 2).

### Propensity score matching analysis

**Figure 1:**
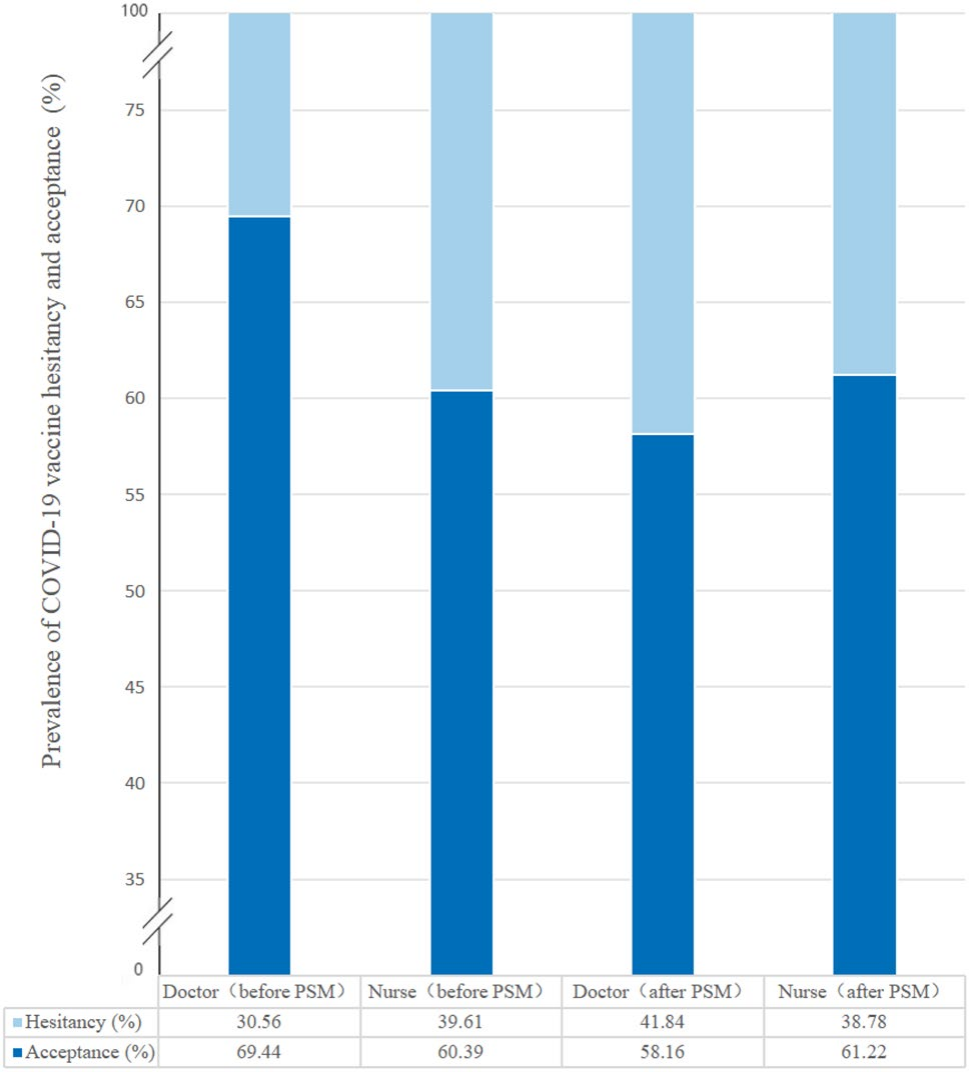
The prevalence of COVID-19 vaccine hesitancy and vaccine acceptance between doctor and nurse in pre- and post-PSM.

**Figure 2:**
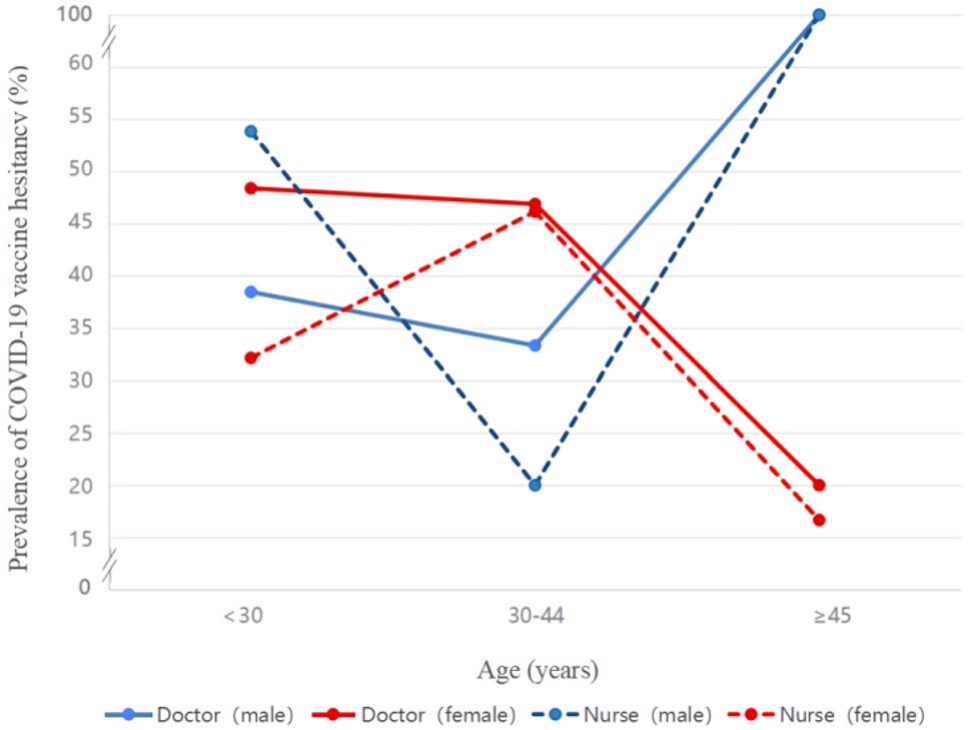
The prevalence of COVID-19 vaccine hesitancy in all age groups by sex between doctor and nurse in post-PSM.

Among 3269 participants, there were 576 doctors and 1068 nurses, resulting in a total of 196 samples that were matched using PSM. After PSM for gender, age, education degree, professional title, health status, and vaccination booster doses, no statistically significant discrepancies could be discerned between the doctor and nurse participants in all covariates (all *P* values > 0.05) (balance test of PSM for doctor and nurse samples was shown in Supplementary file: Table S1). Based on the balanced samples, Fig. 1 showed the difference in COVID-19 vaccine hesitancy between doctor and nurse, illustrating that the prevalence of COVID-19 vaccine hesitancy of nurse (38.78%, 95% CI [28.96%–48.59%]) was very similar to that of doctor (41.84%, 95% CI [31.90%–51.78%]) (*P* = 0.15). In addition, among both doctor and nurse, women were more hesitant than men in the 30–44 age group to get the COVID-19 vaccine, while men were more hesitant in the ≥ 45 age group (Fig. 2).

### Potential reasons of vaccine acceptance and hesitancy

**Figure 3:**
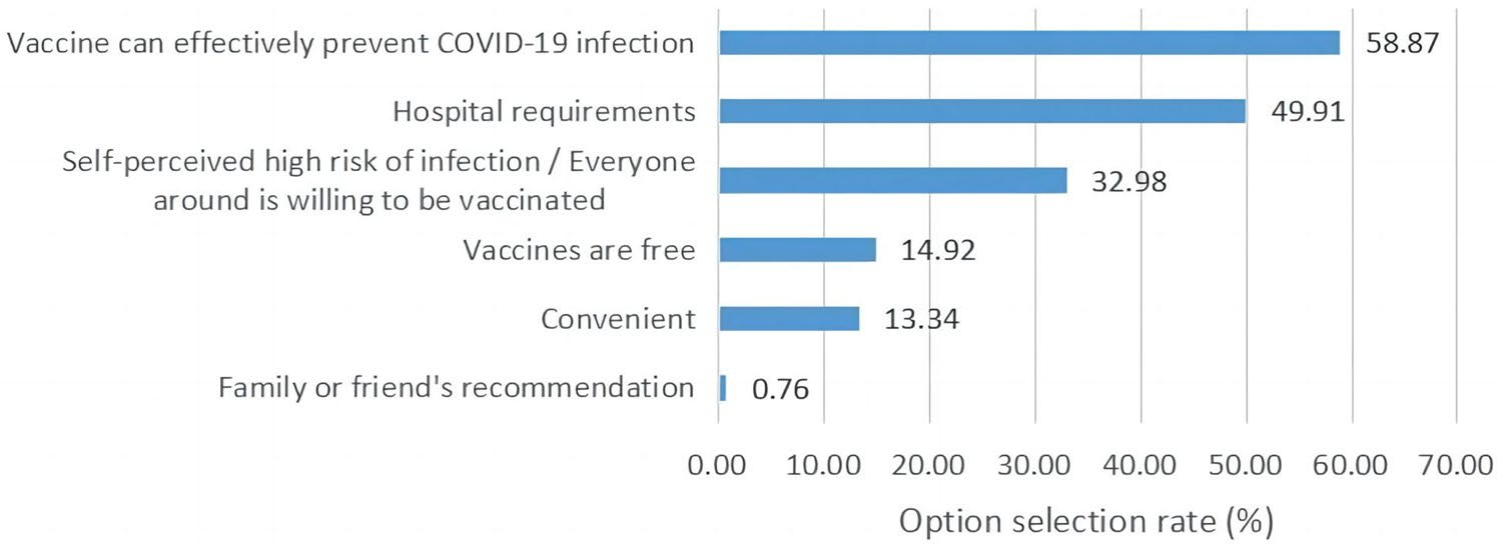
The selection rate of different reasons for vaccine acceptance.

Figure 3 showed all kinds of potential reasons why hospital staff were willing to receive the COVID-19 vaccine. 61.2% of respondents chose only one item of reasons, and 9.1% chose four or more items of reasons. “Vaccine can effectively prevent COVID-19 infection” was the most selected item among the reasons, which 58.87% of staff chose. Other items, such as “Hospital requirements” and “Self-perceived high risk of infection/Everyone around is willing to be vaccinated”, were following, which less than 50% of staff chose. The least selected item was “Family or friend’s recommendation”, which only 0.76% of staff chose.

**Figure 4:**
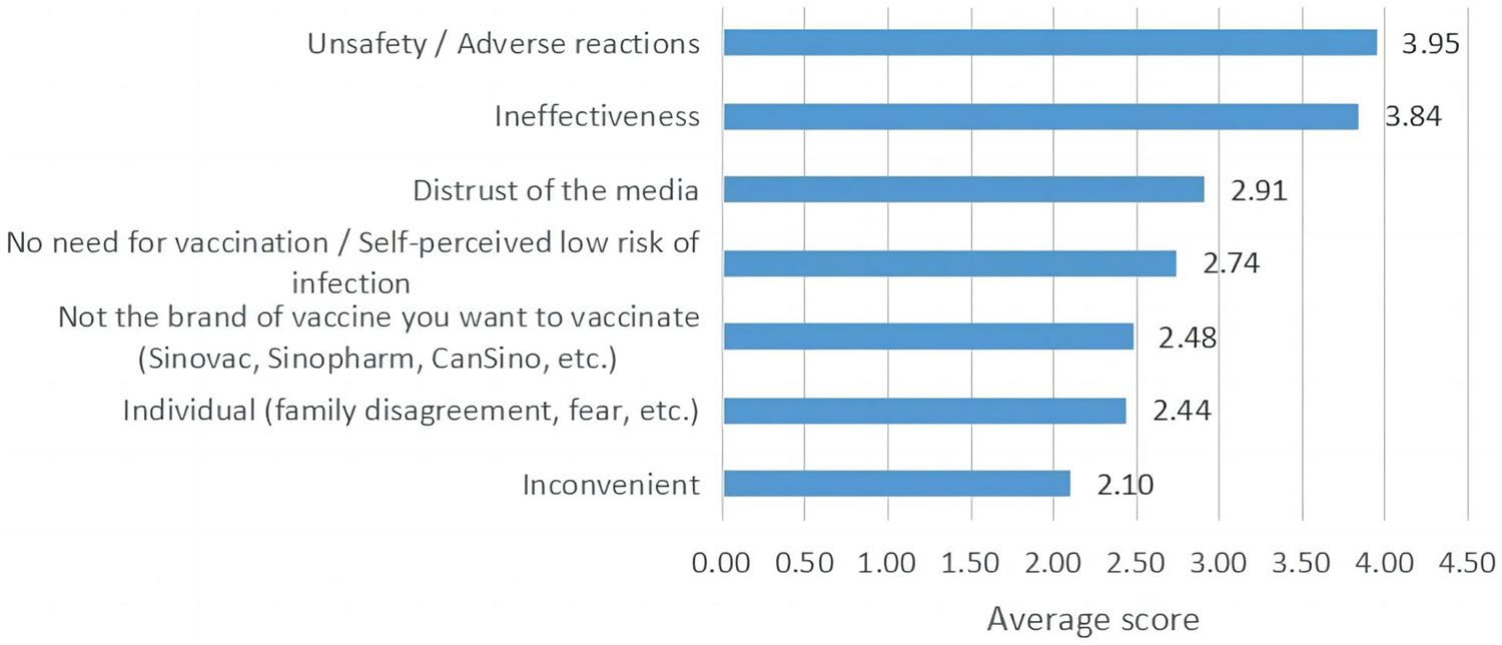
The average score of different reasons for vaccine hesitancy.

Figure 4 showed average scores of respondents at what extent recognizing the reasons for vaccine hesitancy. The item “Unsafety/Adverse reactions” got the highest score (3.95), and the item “Inconvenient” had the lowest score (2.10) among all respondents.

In addition, we found there was no statistically significant difference in the reasons for vaccine hesitancy between the non-vaccinated and vaccinated staff (*P* = 0.844) (data not shown).

## Discussion

Our study found that the rate of COVID-19 vaccine hesitancy of staff in a hospital in Beijing was 32.67%, which was lower than that (47.80%) during the COVID-19 pandemic in 2020^7^ and that (35.50%) after the first round of national COVID-19 vaccination in Chinese general population^20^. A survey of hospital staff in January, 2023 in China showed that 42.20% of healthcare workers self-reported hesitancy about the second dose of COVID-19 vaccine booster^21^. In addition, COVID-19 vaccine hesitancy also exists in some specific groups, for example, among college students, 22.19% of 631 students were hesitant to receive the vaccine^22^. Intriguingly, we found that the attitude of hospital staff towards vaccination was consistent regardless of their jobs, and there was no statistically significant difference, indicating that hospital staffs were probably aware of the similar risk of virus infection in the hospital for everyone.

In the study, we found that women were more likely to hesitate to vaccinate than men, which was consistent with previous findings that women might be more worried about vaccine safety^18,23,24^. The higher level of vaccine hesitancy among women in our study was also reflected in the fact that they showed more concern about the safety of vaccines. On the item “Safety/Adverse reactions”, 81.21% of women agreed or strongly agreed, compared to 68.02% of men. Moreover, we did not find statistically significant difference in vaccine hesitancy among age groups (*P* > 0.05), which differed from previous findings that lower vaccine hesitancy occurred among younger people^25,26^. For one thing, the current policy of public health to prevent and treat severe infections among middle-aged and older adults in China might increase their willingness to vaccinate^27^, and for another, the young people's urgent desire to resume normal life become a main driving force for them to receive the COVID-19 vaccine^28^.

However, when examining the role of gender and age in COVID-19 vaccine hesitancy between doctor and nurse participants after PSM, we found that women in the age group of 30–44 years were more hesitant than men to receive the COVID-19 vaccine, and men aged ≥ 45 years were more hesitant than women among both doctor and nurse. The reason for this might be that social roles and gender roles cause some influence in the attitudes, beliefs and behaviors of individuals. At ages 30–44, women tend to be more stressed about family responsibilities than men and may be more cautious and hesitant about new medications and vaccines. Moreover, previous studies have shown that males and older age were risk factors for COVID-19 deaths^29^. Among older males, there may exist more concerns and worries about vaccine side effects and long-term effects due to possessing a higher risk of death. When these populations perceive that vaccines may have side effects, the risk perception may cause them to be more cautious and show skepticism and doubt about the vaccine.

Furthermore, we found that hospital workers with higher education level had more vaccine hesitancy, which was inconsistent with most existing results that the acceptance of the COVID-19 vaccine increased with income and education level^23,26,30^. However, intriguingly, a study in the United Kingdom found that education was positively associated with vaccine hesitancy after controls for variables^31^; even further studies on the associations between different factors and vaccine hesitancy under different education levels are warranted in the future^32^.

Our study also found that having underlying diseases was associated with higher vaccine hesitancy (*P* = 0.004). Previous studies pointed out that people with underlying diseases might pay more attention to the safety, risks, and adverse side effects of vaccines, and preferred vaccination only for ensuring physical conditions not worse under the vaccine of good quality^33^. Moreover, although most people with underlying diseases acknowledged their increased apprehension about SARS-CoV-2 infection and the importance of the COVID-19 vaccination due to their underlying disease, some of them were worried about vaccine efficacy or the side effect of the vaccine on their current underlying disease^34^.

The items “Safety/Adverse reactions” and “Effectiveness” were most selected in the scale of Reason for Vaccine Hesitancy, which were consistent with findings in existing studies^13,15,35^. Around 57.3% of people who hesitated about the COVID-19 vaccination were worried about the side effects of the COVID-19 vaccine in a study in the United States^36^; even many medical workers would receive COVID-19 vaccination, once much more information about the safety and effectiveness of vaccine was disclosed^37^. As well, most people in China were in a wait-and-see or skeptical attitude towards vaccines at the initial market of vaccines in COVID-19. It is worth noting that the item “Convenient” had a low score in our study, which might be attributed to the rationalization of vaccination services in China, such as extending the duration of vaccination services on weekdays, additionally providing vaccination services on weekends, and adding more vaccination sites.

Whether medical workers have been vaccinated and whether they recommend vaccines to patients have been proven to be important determinants of people’s vaccine acceptance^38^. From a moral point of view, medical workers are responsible and obligated to play a role in COVID-19 vaccination for the public and other hospital staff^39^. In order to improve the vaccine acceptance of hospital staff, comprehensive vaccine knowledge popularization and medical staff training should be carried out, and strategic design should also be carried out at the national level to eliminate misunderstanding of vaccines and improve people's willingness to vaccinate^40^. Medical professionals, as trusted authorities, should actively organize health education and communication to transparently disseminate information about vaccine effectiveness and adverse events and combat disinformation and misinformation^41,42^. In addition, medical professionals should be encouraged to share their stories of vaccination in COVID-19 with relatives, friends, patients and other people around them to build people's confidence and trust in vaccine, and eliminate vaccine hesitancy.

By the analysis of the reasons for acceptance and hesitancy of the COVID-19 vaccine, we can learn about the individual’s attitude and perception related with vaccination, and their vaccine hesitancy behaviors. The study provided evidence supports and supplements to the theory of planned behavior by which most previous studies investigated vaccination intention against COVID-19^43^, thereby could help public health policy makers and vaccine promoters increase the understanding of individual behavioral decision-making and provide more accurate predictions and intervention strategies.

Although the COVID-19 pandemic seems over, the challenge of respiratory infectious disease on the healthcare system still remains. When faced with other emerging infectious diseases, we should realize the importance of education and communication with people learned from this study, to timely communicate the safety and importance of vaccines, and to enhance the accuracy and transparency of information dissemination in case of the spread of false information that confuses the public’s perception of infectious diseases and vaccination likely resulting in vaccine hesitancy.

Our study has some limitations. The participants of the study were staff of a single hospital in Beijing, not involving staff in multi-centers, which might affect the representativeness of subjects and the generalization of findings. Moreover, the online questionnaire was a self-filled questionnaire which might lead to errors in the information by wrong memory and judgment of respondents, accordingly we have adopted quality control to reduce information bias. Furthermore, we did not involve qualitative analyses in our quantitative study, which could provide deeper insights into the reasons behind vaccine hesitancy.

## Conclusions

This study investigated hospital staff's willingness to vaccination against COVID-19 and potential influencing factors associated vaccine hesitancy. In the post-epidemic era, hospitals should strengthen the publicity of vaccine-related knowledge in COVID-19 through internal management, media and social platform to improve hospital staff's attitude and willingness to vaccinations, and thus reduce their vaccine hesitancy, which will further improve vaccination rate, strengthen individual protection and limit the spread of virus. Furthermore, medical workers can be encouraged to proactively correct the misinformation in the public domain about vaccination from a professional point of view, so as to help realize a goal of universal vaccination for COVID-19.

### Supplementary Information

Supplementary Table S1.

## Data Availability

The datasets generated and/or analysed during the current study are not publicly available due to confidentiality agreements with the Administration Office of Science and Technology of Beijing Hospital but are available from the corresponding author on reasonable request.

## Abbreviations

COVID-19: Novel coronavirus disease
PSM: Propensity score matching
SAGE: The Strategic Advisory Group on Experts
