# Supplementary material for: COVID-19 vaccine hesitancy and influencing factors among Chinese hospital staff: a cross-sectional study
Source: Sci Rep. 2024 Feb 22;14:4369. doi: 10.1038/s41598-024-55001-z (PMC10883913; doi:10.1038/s41598-024-55001-z)
Supplement: Supplementary file 1 — Supplementary Table S1. [file 41598_2024_55001_MOESM1_ESM.docx]

**Table S1.** Balance test of PSM for doctor and nurse samples

| Variables | Pre-PSM SMD | Post-PSM SMD |
| --- | --- | --- |
| Gender | 0.260 | 0.019 |
| Age | 0.133 | 0.048 |
| Education degree | 0.128 | 0.009 |
| Professional title | 0.239 | 0.040 |
| Health status before infection | 0.096 | 0.041 |
| Vaccination Booster Doses | 0.422 | 0.030 |

PSM, Propensity Score Matching; SMD, Standardized mean difference
